# INTERDISCIPLINARY WELLNESS CLINICS IN LOW-INCOME SENIOR HOUSING COMMUNITIES

**DOI:** 10.1093/geroni/igad104.0598

**Published:** 2023-12-21

**Authors:** Sarah Holmes

## Abstract

Low-income older adults may qualify to live in subsidized housing via the Housing and Urban Development (HUD) Section 202 program. Established in 1959, Section 202 is the only HUD program that provides housing exclusively for older adults. More than 1.8 million older adults receive this type of federal housing assistance. Older adults living in low-income senior housing communities are mostly people of color, socioeconomically disadvantaged, and at risk for poor physical and mental health and adverse health outcomes. Additionally, they have limited access to healthcare services and other resources such as internet use, healthy food options, opportunities for physical activity, and safe indoor and outdoor physical environments. Further they are at increased risk of high emergency room utilization and transfer to nursing home settings. The purpose of this project was to develop Interdisciplinary Wellness Clinics for older adults living in low-income senior housing communities and provide direct services including such things as blood pressure monitoring, medication management, hearing evaluation and cerumen removal, foot and nail care, pain management, management of acute medical problems, immunizations, and Medicare Annual Wellness Visits. Interdisciplinary Wellness Clinics are provided monthly to four low-income senior housing communities and serve approximately 500 residents living in these communities. This symposium will provide a review of residents seen and services provided with a focus on findings identified and interventions implemented; a description of the Medicare Annual Wellness services; and a review of approaches used to engage residents in using these services.

